# The management of tetanus in adults in an intensive care unit in Southern Vietnam

**DOI:** 10.12688/wellcomeopenres.16731.1

**Published:** 2021-05-12

**Authors:** Nguyen Van Hao, Lam Minh Yen, Rachel Davies-Foote, Truong Ngoc Trung, Nguyen Van Thanh Duoc, Vo Thi Nhu Trang, Phung Tran Huy Nhat, Du Hong Duc, Nguyen Thi Kim Anh, Pham Thi Lieu, Tran Thi Diem Thuy, Duong Bich Thuy, Nguyen Thanh Phong, Nguyen Thanh Truong, Pham Ba Thanh, Dong Thi Hoai Tam, Zudin Puthucheary, C Louise Thwaites

**Affiliations:** 1 Hospital for Tropical Diseases, Ho Chi Minh City, Vietnam; 2 University of Medicine and Pharmacy, Ho Chi Minh City, Vietnam; 3 Oxford University Clinical Research Unit, Ho Chi Minh City, Vietnam; 4 London School of Hygiene & Tropical Medicine, London, UK; 5 Gia Dinh Hospital, Danang City, Vietnam; 6 William Harvey Research Institute, Queen Mary, University of London, London, UK; 7 Royal London Hospital, London, UK; 8 Centre for Tropical Medicine and Global Health, University of Oxford, Oxford, UK

**Keywords:** Tetanus, management, treatment, low middle income country, LMIC, intensive care unit, ICU

## Abstract

**Background:** Tetanus remains common in many low- and middle-income countries (LMICs) yet the evidence base guiding management of this disease is extremely limited, particularly with respect to contemporary management options. Sharing knowledge about practice may facilitate improvement in outcomes elsewhere.

**Methods**: We describe clinical interventions and outcomes of 180 adult patients ≥16 years-old with tetanus enrolled in prospective observational studies at a specialist infectious diseases hospital in Southern Vietnam. Patients were treated according to a holistic management protocol encompassing wound-care, antitoxin, antibiotics, symptom control, airway management, nutrition and de-escalation criteria.

**Results**: Mortality rate in our cohort was 2.8%, with 90 (50%) patients requiring mechanical ventilation for a median 16 [IQR 12-24] days. Median [IQR] duration of ICU stay was 15 [8-23] days.  Autonomic nervous system dysfunction occurred in 45 (25%) patients. Hospital acquired infections occurred in 77 (43%) of patients.

**Conclusion**: We report favourable outcomes for patients with tetanus in a single centre LMIC ICU, treated according to a holistic protocol. Nevertheless, many patients required prolonged intensive care support and hospital acquired infections were common.

## Introduction

Tetanus is a vaccine-preventable disease that remains a common cause of acute critical illness in low-income and middle-income countries (LMICs) ^1^. Signs and symptoms are due to the effects of tetanus toxin in the central nervous system and management is based on three key strategies: blocking further tetanus toxin release ^2^, neutralising unbound toxin ^3^, and alleviating effects of already-bound toxin; namely muscle spasms and autonomic nervous system dysfunction ^1, 4, 5^. With access to critical care interventions such as mechanical ventilation and advanced physiological monitoring, muscle spasms and autonomic nervous system dysfunction (ANSD) can be more easily managed ^6– 8^. These interventions are now available in many LMIC intensive care units (ICUs); however, their availability is often not associated with improved outcomes ^9, 10^.

As almost all tetanus occurs in settings with limited capacity for clinical trials, the evidence base for tetanus management remains limited. There are few randomized clinical trials to support common management strategies and, in the absence of high-quality evidence, observational studies and case series become the key elements in guiding treatment. The Hospital for Tropical Diseases, Ho Chi Minh City, has been a tertiary referral centre for tetanus for over 30 years and has developed and implemented a specific holistic management protocol for patients with tetanus. The ICU continues to admit several hundred adult patients with tetanus every year and reports outcomes comparable with those from high income settings ^7, 11^.

The overall aim of this paper is to pragmatically describe the intensive care management of adult tetanus in a LMIC setting but nevertheless one with amongst the lowest reported case fatality rate worldwide ^12^.

## Methods

### Setting

**Figure 1.**
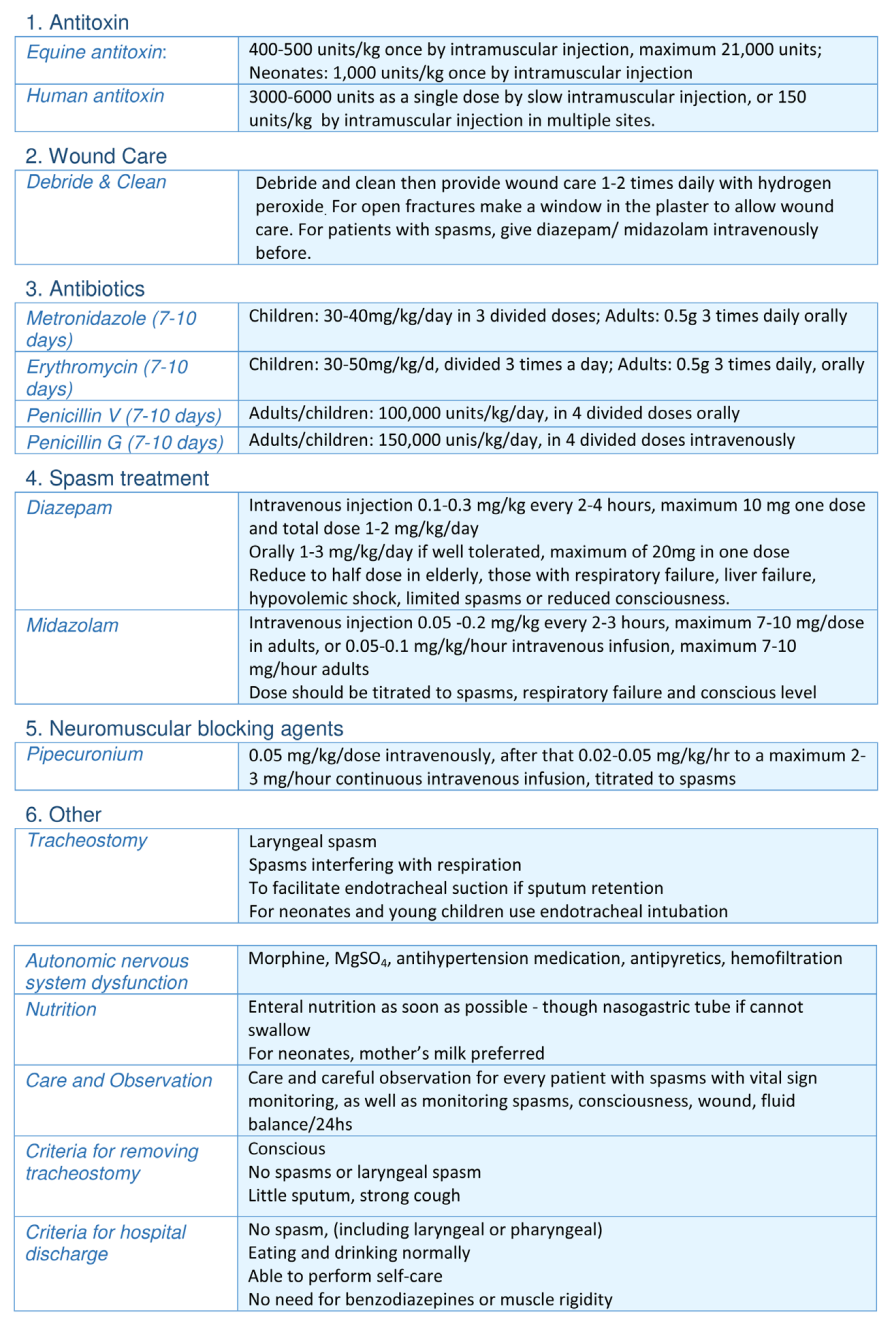
Summary of Tetanus Treatment Protocol Hospital for Tropical Diseases, Ho Chi Minh City.

The Hospital for Tropical Diseases (HTD), Ho Chi Minh City is a tertiary referral centre for infectious diseases serving Southern Vietnam. Previously the hospital housed a special tetanus ICU but whilst this no longer exists, the hospital’s adult ICU continues to receive 250-350 adult patients with tetanus annually. The principles of tetanus management described above have been incorporated into a specific treatment protocol ( Figure 1), which has been applied consistently to all patients over a 10 year period ^12^. In addition to pharmacological interventions, the protocol includes directions for airway management, nutrition and nursing observations. It also includes criteria for de-escalation and discharge from hospital.

### Participants and data collection

Data on management and outcome of patients treated with this protocol were collected from prospective observational studies of two cohorts of patients ≥16 years old admitted to the hospital’s ICU with a diagnosis of generalized tetanus; the first from August 2016 - March 2017 and the second from January - July 2018. For patients enrolled between August 2016 and March 2017, additional exclusion criteria were: (i) not speaking Vietnamese, (ii) not being able to walk before admission.

Baseline and clinical variables including patient demographic details, tetanus severity indicators and management interventions and complications were collected prospectively on all enrolled subjects. Enrolled patients were followed daily until hospital discharge. Previously described definitions were applied for hospital acquired infections ^13^. Autonomic nervous system dysfunction (ANSD) was defined as at least three of: heart rate >100 beats per minute (bpm), systolic blood pressure >140 mmHg, mean arterial pressure < 60 mmHg, pyrexia > 38°C, and fluctuating blood pressure. All features should be present within one day with no other apparent cause ^14^. Sensitivity evaluation of mortality rates within this study was performed by comparing with overall hospital database for outcomes of all patients with tetanus (ICD10 code A35) during the period 2016- 2018.

### Statistical analysis

Descriptive statistics were used to describe the sample with the median and interquartile range (IQR) for continuous data, and count and percentage for categorical data. Due to small numbers of those who died, no comparative statistics have been performed. All analyses were carried out in Stata (StataCorp) version 16. Missing data are included and described in tables.

### Ethics statement

This study was approved by the London School of Hygiene and Tropical Medicine (LSHTM) ethics committee, the Oxford Tropical Medicine Ethics Committee (OxTREC) and the local HTD ethics committee (Refs 16904, 596-16, 816 QD-BVBND, 38-17, 494/ QD – SYT respectively.) All participants gave written informed consent to participate before enrolment.

## Results

In total, 180 patients with generalised tetanus admitted to the ICU at HTD between August 2016 and July 2018 were included in this study. Out of a total 160 adult patients with tetanus admitted to the ICU, 80 patients were enrolled during the first period. For the second period, 100 patients were included out of a total of 120 admissions. Reasons for lower enrolment of the first cohort were largely pragmatic due to lack of availability of study staff and more stringent enrolment criteria. The median [IQR] age of the patients was 51.0 [40.8-61.5]. The youngest age was 17 and the oldest 98 years old. Of 180 patients, 73 (40.6%) had at least one comorbidity and 143/180 (79.4%) were male. Median Tetanus Severity Score on admission was 1.5 [IQR -3 – 5] ^15^. Severe tetanus, defined as Ablett grade 3 or 4 on hospital admission (i.e. spasms interfering with respiration with/without autonomic nervous system dysfunction), was diagnosed in 28 patients (16%), but an additional 66 (37%) progressed to severe disease during hospitalization. 

A summary of the management and complications of the patients during ICU admission are described in Table 1 and Table 2.

**Table 1.** Description of intensive care unit management of enrolled tetanus patients.

Parameter	Median [IQR] or Count (%)
*Interventions*	
**Tracheostomy required**	94 (52.2%)
**Duration tracheostomy (days) (n=94)**	18.5 [15-27]
**Mechanical ventilation required**	90 (50%)
**Duration mechanical ventilation (days) (n=90)**	16.0 [12-24]
*Pharmaceutical agents required*	
**Duration diazepam required (days) (n=174)**	14 [11-20]
**Total dose diazepam (mg) (n=174)**	585 [295-1352.5]
**Maximum dose in 24 hours diazepam IV (mg) (n=97)**	80 [20-120]
**Maximum dose in 24 hours diazepam oral (mg) (n=170)**	60 [45-120]
**Maximum dose in 24 hours diazepam any route (mg) (n=173)**	120 [60-120]
**Duration midazolam required (days) (n=109)**	10 [3-16]
**Total dose midazolam during hospitalization (mg) (n=109)**	996 [240-1905]
**Maximum dose in 24 hours midazolam (mg) (n=109)**	120 [92-168]
**Total dose benzodiazepine during hospitalization (mg)**	1627.5 [862.5-2526.2]
**Maximum dose in 24 hours benzodiazepine (mg)**	120 [120-160]
**Duration magnesium sulphate (days) (n=50)**	5 [3-8]
**Total dose magnesium sulphate during hospitalization (g)** **(n=50)**	203 [81-336]
**Duration pipecuronium (days) (n=78)**	13 [9-17]
**Total dose pipecuronium during hospitalization (mg) (n=78)**	410.3 [226.3-558.3]

**Table 2.** Complications and outcomes of enrolled patients.

Parameter	Median [IQR] or Count (%)
*Severity score*	
Worst Ablett score during admission *: **1** **2** **3** **4**	20 (11.1%) 66 (36.7%) 49 (27.2%) 45 (25%)
*Complications*	
**Autonomic nervous system dysfunction**	45 (25%)
**Ventilator associated pneumonia**	57 (31.7%)
**Bacteraemia**	19 (10.6%)
**Urinary tract infection**	39 (21.7%)
**Any hospital acquired infection**	77 (42.8%)
**Pressure ulcer**	18 (10%)
***Duration of admission***	
**Length of intensive care unit stay (days)**	15.0 [8.0-23.0]
**Length of hospital stay (days)**	25 [19.0-34.0]
*Outcome*	
Died in hospital **	5 (2.8%)

*Ablett score: Grade 1 is mild tetanus with no spasms, Grade 2 mild spasms not compromising breathing, Grade 3 is severe tetanus with spasms compromising breathing. Ablett Grade 4 is as Grade 3 but with additional signs of autonomic nervous system dysfunction ^21^. ** Includes those taken home, expected to die.

### Description of the cases who died

Of the 5/180 (2.8%) patients that died, the median age was 79 years [IQR 84-81], compared with 50 years [IQR 40-61] for the 175/180 (97.2%) patients who survived; 5/5 (100%) were male and 4/5 (80%) had comorbidities. On admission, amongst those who died, all 5/5 (100%) had difficulty breathing (noted by admitting doctors) compared to 32/175 (18.3%) for those who survived. The median SpO _2_ was 94% [IQR 94-97] compared with 97% [IQR 95-98] for those who survived and the median white blood cell (WBC) count was 16.8 [14.9-23.9] × 10 ^9^/L compared with 9.8 [7.4-11.8] × 10 ^9^/L for those who survived. Throughout ICU admission, amongst those who died, all 5/5 (100%) required mechanical ventilation. Two out of five (40%) patients that died developed ANSD, compared with 43/175 (24.6%) in those who survived.

Three deaths were caused by cardiogenic shock (occurring at days 2, 12 and 15 of ICU admission), one death was due to septic shock secondary to ventilator associated pneumonia (occurring at day 5 of ICU admission) and one was due to ischaemic bowel and perforation (occurring at day 28 of ICU admission). Review of hospital records showed that in total, during the three years 2016- 2018, 917 adults were admitted with tetanus with an overall case fatality rate of 4% (including palliative discharges).

## Discussion

We describe clinical features and outcomes of a large cohort of patients with tetanus managed at a specialist tetanus centre. Patients were managed in accordance with a standardized protocol by a team of doctors and nurses with significant experience in tetanus management ^7^.

The case fatality rate in this study is 2.8%. This is, to our knowledge, the lowest reported mortality rate for a large series of tetanus patients worldwide ^12^, and contrasts with rates reported from many other LMICs ^10, 16^. Whilst it is possible that selection bias has influenced our results, our figures are similar to official hospital records over the study period as well as an observational study enrolling patients with severe tetanus from our centre and one other major centre in Vietnam between 2013 and 2015 ^17^. Comparison with contemporary data from other countries is more difficult due to limited reporting of established tetanus severity scores or known prognostic features. Nevertheless, the age of patients in our study (one of the strongest predictors of outcome) is similar to, or even older than, those reported in other centres with worse outcomes ^10, 18, 19^. Similarly, our ventilation rate was 50% but rates between 50% and 75% elsewhere have been associated with mortality rates of 30–35% ^10, 19, 20^.

We believe that the favourable outcomes at our centre result from two major factors: a clear evidence-based management protocol and care by a highly specialized team with enormous experience in tetanus. Throughout the world, protocolized medical care is encouraged as a means of improving patient outcomes. Ideally, protocols are based on best evidence and can be regularly updated. However, this is not the case for many of the elements of our protocol due to the lack of high-quality contemporary evidence for tetanus management. Nevertheless, the outcomes in our patients to some extent supports their continued use. A limitation of our work is that protocol adherence itself was not specifically measured. Personal experience and treatment intervention data reported herein indicate that adherence was high; however, we have not specifically examined compliance with individual components of the protocol.

Our hospital is a tertiary infectious disease centre and receives patients with tetanus from Southern Vietnam. A highly developed referral system and limited staff turnover within the ICU means that experience in management of tetanus can be more readily easily developed and preserved. Tetanus is a disease where progression continues to occur after hospitalization. Experienced staff may therefore be better able to anticipate complications, and so arrange care and interventions more appropriately. They may also be able to pass on subtle elements of care not outlined in our protocol – for example exactly when to intervene with spasms or how to balance risk of pressure area necrosis and spasm provocation when turning a patient. Finally, as a tertiary infectious disease centre there may be further factors that particularly benefited outcomes, such as more careful prevention or management of hospital acquired infections which are particularly frequent in severe tetanus.

Sharing these forms of tacit knowledge is a challenge for health systems across the world but is most likely to benefit lower resourced settings with less access to specialised training and referral. The current expansion of digital technologies may offer possible solutions. For example, newer technologies in the form of telemedicine or even AI-enabled risk stratification may facilitate dissemination of less explicit knowledge or even simplify analysis of these complex processes.

## Conclusions

We report management and outcome features in a large contemporary cohort of patients with tetanus treated according to a standardized protocol. Survival rates of these patients are high compared to other reported case series. Nevertheless, other outcomes such as duration of hospitalization and mechanical ventilation requirements indicate that tetanus remains a significant burden on healthcare services. Therapies that can reduce these continue to be needed.

## Declarations

### Data availability

Oxford University Research Archive. Dataset: Long-term outcome in tetanus cohort: 04TS: https://ora.ox.ac.uk/objects/uuid:af44c622-a7b8-44b2-827e-056623dd49a8 ^22^.

This project contains the following underlying data:

- ORA_04TSSF36.xlsx (This is a data set from a clinical observation study. The data was manually entered from case record forms to a specific database. This dataset is from the exported data.)

Data are available under the terms of the Creative Commons Attribution 4.0 International license (CC-BY 4.0).
