# Supplementary material for: The management of tetanus in adults in an intensive care unit in Southern Vietnam
Source: Wellcome Open Res. 2021 Aug 31;6:107. Originally published 2021 May 12. [Version 2] doi: 10.12688/wellcomeopenres.16731.2 (PMC8185581; doi:10.12688/wellcomeopenres.16731.2)
Supplement: Peer review history

# Peer review: Reviewer response for version 2

**DOI:** 10.21956/wellcomeopenres.18786.r45681

**Published:** 2021-09-07

**Authors:** Bharath Kumar Tirupakuzhi Vijayaraghavan

**Affiliations:** 1 Department of Critical Care Medicine, Apollo Hospitals, Chennai, Tamil Nadu, India

All my comments have now been addressed. I have nothing further to add. 

Is the work clearly and accurately presented and does it cite the current literature?

Yes

If applicable, is the statistical analysis and its interpretation appropriate?

Not applicable

Are all the source data underlying the results available to ensure full reproducibility?

Yes

Is the study design appropriate and is the work technically sound?

Yes

Are the conclusions drawn adequately supported by the results?

Yes

Are sufficient details of methods and analysis provided to allow replication by others?

Yes

Reviewer Expertise:

My clinical and research area of interest is broadly critical care - I also have specific experience in the management of critically ill patients with tetanus.

I confirm that I have read this submission and believe that I have an appropriate level of expertise to confirm that it is of an acceptable scientific standard.

# Peer review: Reviewer response for version 2

**DOI:** 10.21956/wellcomeopenres.18786.r45682

**Published:** 2021-09-07

**Authors:** Ana Bonell

**Affiliations:** 1 MRC Unit The Gambia, London School of Hygiene and Tropical Medicine, Fajara, The Gambia; 2 Centre on Climate Change and Planetary Health, London School of Hygiene and Tropical Medicine, London, UK

I am happy to accept the article as is and have no new comments.

Is the work clearly and accurately presented and does it cite the current literature?

Yes

If applicable, is the statistical analysis and its interpretation appropriate?

Yes

Are all the source data underlying the results available to ensure full reproducibility?

Yes

Is the study design appropriate and is the work technically sound?

Yes

Are the conclusions drawn adequately supported by the results?

Yes

Are sufficient details of methods and analysis provided to allow replication by others?

Yes

Reviewer Expertise:

NA

I confirm that I have read this submission and believe that I have an appropriate level of expertise to confirm that it is of an acceptable scientific standard.

# Peer review: Reviewer response for version 1

**DOI:** 10.21956/wellcomeopenres.18452.r43840

**Published:** 2021-06-07

**Authors:** Madiha Hashmi

**Affiliations:** 1 Department of Critical Care Medicine, Ziauddin University, Karachi, Pakistan

Favorable outcomes of adult patients with generalized tetanus are attributed to consistent, holistic, evidence-based and protocolized management by a highly experienced team at a specialist infectious diseases hospital in Southern Vietnam that receives 250-350 adult patients annually.

 This prospective observational study describes the pragmatic management and outcomes of 180 patients in two cohorts (August 2016 - March 2017 and January - July 2018) that were followed daily till hospital discharge. Factors like age, need for mechanical ventilation (50%), autonomic nervous system dysfunction (25%), hospital acquired infections (43%) and median length of ICU stay (15 [8-23] days) is comparable to reports from other LMICs but outcome is significantly better (2.8% vs. 44% from a Brazilian ICU).

**Study Design:**

- The prospective observational study design describes better outcomes of adult patients admitted with generalized tetanus based on a high volume of cases referred to this tertiary center and more than 10 years experience of the team rather than adherence to the elements of the management protocol. Randomization of patients to either receive a bundled care or usual care or studying outcomes based on adherence to the holistic protocol will improve the quality of evidence generated.
- The full exclusion criteria are not mentioned. It is not clear why ALL patients admitted during the two study periods were not included in the study and 80 patients (out of 160) in the first study period and 20 (patients (out of 120) in the second study period were excluded.

**Reproducibility:**

- To allow others to benefit from their holistic protocols more details of various elements of the protocol are needed like recommended timings of antitoxins, antibiotics, tracheostomy or mechanical ventilation, details of wound management, targets of nutrition therapy, infection prevention and control protocols, routine prophylactic therapies and rehabilitation protocols, etc.

Is the work clearly and accurately presented and does it cite the current literature?

Yes

If applicable, is the statistical analysis and its interpretation appropriate?

Yes

Are all the source data underlying the results available to ensure full reproducibility?

Partly

Is the study design appropriate and is the work technically sound?

Partly

Are the conclusions drawn adequately supported by the results?

Yes

Are sufficient details of methods and analysis provided to allow replication by others?

Yes

Reviewer Expertise:

surgical intensive care, anesthesia, critical care epidemiology

I confirm that I have read this submission and believe that I have an appropriate level of expertise to confirm that it is of an acceptable scientific standard.

# Peer review: Reviewer response for version 1

**DOI:** 10.21956/wellcomeopenres.18452.r43839

**Published:** 2021-06-01

**Authors:** Bharath Kumar Tirupakuzhi Vijayaraghavan

**Affiliations:** 1 Department of Critical Care Medicine, Apollo Hospitals, Chennai, Tamil Nadu, India

Thanks to the authors for this important submission.

 Dr Van Hao and colleagues describe their experience and the management of 180 adult patients with tetanus from a single-ICU from Southern Vietnam.  As pointed out by the authors, tetanus remains a vaccine preventable, but neglected disease in several LMICs. Data on management strategies and outcomes from high-performing centers is not only critical for guiding other hospitals in the region and from other LMICs, but also in setting the research agenda. 

 I have a few comments for the authors to consider:

**Major comments:**

1. Why is there a gap in the study periods? Is this due to the unavailability of data in the intervening period? Or is it just a reflection of the fact that the authors use data from two readily available prospective cohorts (where information was collected for other reasons)?
  A related question is if outcomes varied between the periods as well in the intervening period ( I recognize that the overall mortality is low and is perhaps unlikely to be very different between periods).
2. The outcomes are impressive - is this due to early identification and referral? Could the authors comment on this- perhaps provide information on time from symptom onset to admission?
3. Do the authors have information on intermediate-long term outcomes (survival, QoL, etc.) for these patients? 
4. The authors provide data on nosocomial infections- how do these rates compare to the non-tetanus intensive care admissions? And how do the outcomes for nosocomial infections compare?  Do they have information on the pathogen profile (for instance proportion of multidrug resistant organisms)?
5. In their treatment protocol, I presume several of the treatments listed are 'OR' options rather than 'AND'- for example equine **or** human antitoxin, diazepam **or** midazolam etc. Can they clarify this in the table so that it is obvious to the readers?
  I know this is not listed in their protocol- but are any intrathecal therapies used?
6. The section on 'description of cases who died': I agree that numbers are small, but the authors could provide the information given in this section as a table comparing 'dead' vs 'survivors'- they don't have to perform any hypothesis testing. 
7. Table 2 provides the worst Ablett score during admission- numbers provided in this table do not match with those described under the text (in 'Results').
8. In the Discussion section, the authors could briefly highlight what they see as the research priorities for tetanus given their experience (for example, need for data on long-term outcomes, more stringent evaluation of therapies through trials, need for a registry tracking outcomes, etc.)

**Minor comments:**

1. Under 'Results': Line 3: Why 160 patients? Shouldn't this be 180?
  Line 6: why 100 patients included out of 120- reasons for exclusion of 20?
2. Under 'participants and data collection': what is the justification of the additional exclusion criteria (lines 7,8)? Is it just a carryover of the exclusion criteria of the original cohort?
3. Under 'Discussion': Para 3- the authors say their protocol is 'evidence-based' but then go on to also say that most elements don't have much evidence. I know what they mean- but they could reframe these sentences. 
4. The authors note that for a period these patients were managed in a 'special tetanus ICU'- are there outcomes from that period? and if yes how do they compare to the later period?
5. I note their protocol includes elements around nutrition, discharge criteria, etc. Do they also have a plan for physiotherapy or rehab (in the later phases of illness)- these patients require prolonged periods of sedation/paralysis - hence the question.

Is the work clearly and accurately presented and does it cite the current literature?

Yes

If applicable, is the statistical analysis and its interpretation appropriate?

Not applicable

Are all the source data underlying the results available to ensure full reproducibility?

Yes

Is the study design appropriate and is the work technically sound?

Yes

Are the conclusions drawn adequately supported by the results?

Yes

Are sufficient details of methods and analysis provided to allow replication by others?

Yes

Reviewer Expertise:

My clinical and research area of interest is broadly critical care - I also have specific experience in the management of critically ill patients with tetanus.

I confirm that I have read this submission and believe that I have an appropriate level of expertise to confirm that it is of an acceptable scientific standard, however I have significant reservations, as outlined above.

# Response

**Published:** 2021-06-23

**Authors:** C Louise Thwaites

**Affiliations:** Oxford University Clinical Research Unit, Vietnam

*Why is there a gap in the study periods? Is this due to the unavailability of data in the intervening period? Or is it just a reflection of the fact that the authors use data from two readily available prospective cohorts (where information was collected for other reasons)?*

 The latter is correct. This has been clarified in the text in the methods section.

*A related question is if outcomes varied between the periods as well in the intervening period ( I recognize that the overall mortality is low and is perhaps unlikely to be very different between periods).*

 We have included the overall death rate of 4% in ALL hospital admissions in the results section. This rate includes all the 3 years and all patients (not just those enrolled). As the first cohort excluded those who could not walk, and could potentially have excluded the most severe group of patients as discussed in the Discussion section.

*The outcomes are impressive - is this due to early identification and referral? Could the authors comment on this- perhaps provide information on time from symptom onset to admission?*

 This has been added after the Tetanus Severity Score.

*Do the authors have information on intermediate-long term outcomes (survival, QoL, etc.) for these patients?*

 Long-term outcomes are available in the first cohort of these patients and are published separately. This has been added and cited again.

*The authors provide data on nosocomial infections- how do these rates compare to the non-tetanus intensive care admissions? And how do the outcomes for nosocomial infections compare?  Do they have information on the pathogen profile (for instance proportion of multidrug resistant organisms)?*

 For this study, we have not addressed this specifically. We have in previous studies examined and compared rates of nosocomial infections  - as tetanus patients stay for long periods of time but have relatively little multi-organ failure compared to other patients with similar length of stay, analysis is fairly complex and has not been included here. (See PLoS One. 2018 Sep 7;13(9):e0203600)

*In their treatment protocol, I presume several of the treatments listed are 'OR' options rather than 'AND'- for example equine or human antitoxin, diazepam or midazolam etc. Can they clarify this in the table so that it is obvious to the readers?*

 Thank you – these have been clarified.

*I know this is not listed in their protocol- but are any intrathecal therapies used?*

 No these are not currently used.

*The section on 'description of cases who died': I agree that numbers are small, but the authors could provide the information given in this section as a table comparing 'dead' vs 'survivors'- they don't have to perform any hypothesis testing*.

 This has been done and a table added.

*Table 2 provides the worst Ablett score during admission- numbers provided in this table do not match with those described under the text (in 'Results').*

 The initial results section contains the 28 patients with severe on admission, the table contains the number of patients with severe scores (3 or 4) during admission. The text has been altered to clarify this.

*In the Discussion section, the authors could briefly highlight what they see as the research priorities for tetanus given their experience (for example, need for data on long-term outcomes, more stringent evaluation of therapies through trials, need for a registry tracking outcomes, etc.)*

 Minor comments:

*Under 'Results': Line 3: Why 160 patients? Shouldn't this be 180*?

 This was not clear – 80/160 admissions during the first period were enrolled in this study and 100/120 admissions during the second period were enrolled. The text has been changed.

*Line 6: why 100 patients included out of 120- reasons for exclusion of 20?*

  As above. Entry criteria are given in the methods.

*Under 'participants and data collection': what is the justification of the additional exclusion criteria (lines 7,8)? Is it just a carryover of the exclusion criteria of the original cohort?*

  Yes – text has been altered.

*Under 'Discussion': Para 3- the authors say their protocol is 'evidence-based' but then go on to also say that most elements don't have much evidence. I know what they mean- but they could reframe these sentences*.

 Thank you – we have removed these/ changed to ‘best-available evidence’

*The authors note that for a period these patients were managed in a 'special tetanus ICU'- are there outcomes from that period? and if yes how do they compare to the later period?*

 Yes, we have data – added in the discussion section and cited.

*I note their protocol includes elements around nutrition, discharge criteria, etc. Do they also have a plan for physiotherapy or rehab (in the later phases of illness)- these patients require prolonged periods of sedation/paralysis - hence the question*.

 Currently, these are not written in the protocol but we are aiming to develop these. This has been added to the discussion in the ‘priorities’ and future section.

# Peer review: Reviewer response for version 1

**DOI:** 10.21956/wellcomeopenres.18452.r43838

**Published:** 2021-05-28

**Authors:** Ana Bonell

**Affiliations:** 1 MRC Unit The Gambia, London School of Hygiene and Tropical Medicine, Fajara, The Gambia; 2 Centre on Climate Change and Planetary Health, London School of Hygiene and Tropical Medicine, London, UK

This study presents outcome data for patients with severe tetanus who were treated according to a set treatment protocol developed in an intensive care unit in Vietnam. Data was collected from 2016-2018 and includes 180 patients. Mortality outcomes for this cohort is very low (2.8%) compared to other published studies from LMICs.

 Thank you for the opportunity to review this study detailing your standardised protocol. 

 I have a few minors points for your consideration:

- Is it possible to make figure 1 into a flowchart? This would be easier for clinicians to follow. Or potentially a checklist like the surgical checklist for example so that clinicians can easily see if they have missed any points.
- Results: In the results section, please check IQR of median age of patients that died and check median age – it doesn't look correct.
- I would like to understand the hospital acquired infection rate in context – how does this compare to other conditions on that specific ICU and also compared to other tetanus cohorts (43% is high).
- In the discussion, please can you add  the case fatality rates from other settings for direct comparison (e.g. https://pubmed.ncbi.nlm.nih.gov/33824951/) ^1^

Is the work clearly and accurately presented and does it cite the current literature?

Yes

If applicable, is the statistical analysis and its interpretation appropriate?

Yes

Are all the source data underlying the results available to ensure full reproducibility?

Yes

Is the study design appropriate and is the work technically sound?

Yes

Are the conclusions drawn adequately supported by the results?

Yes

Are sufficient details of methods and analysis provided to allow replication by others?

Yes

Reviewer Expertise:

Anaesthetics and intensive care | climate change and maternal health

I confirm that I have read this submission and believe that I have an appropriate level of expertise to confirm that it is of an acceptable scientific standard, however I have significant reservations, as outlined above.

## References

[1] : Tetanus in a rural low-income intensive care unit setting.*Brain Commun*.2021;3(1) : 10.1093/braincomms/fcab013fcab01310.1093/braincomms/fcab01333824951PMC8010432

# Response

**Published:** 2021-06-23

**Authors:** C Louise Thwaites

**Affiliations:** Oxford University Clinical Research Unit, Vietnam

*Is it possible to make figure 1 into a flowchart? This would be easier for clinicians to follow. Or potentially a checklist like the surgical checklist for example so that clinicians can easily see if they have missed any points*.

 This is a very good suggestion. Currently, the purpose of this piece was to publish in English the protocol which is already in use in the Hospital for Tropical Diseases. However, the idea to create a flow-chart check list is excellent and we will work on defining the action/stop-start points.

*Results: In the results section, please check IQR of median age of patients that died and check median age – it doesn't look correct.~*

 This has been checked and now is included in Table 3

 I *would like to understand the hospital acquired infection rate in context – how does this compare to other conditions on that specific ICU and also compared to other tetanus cohorts (43% is high).*

 In this study, we have not specifically looked at that, however previous studies have examined this. We have included a discussion around this in the discussion section. Essentially what we have found in previous studies is that although rates are high, this is generally due to the length of ICU stay and that outcomes are actually relatively good.

*In the discussion, please can you add the case fatality rates from other settings for direct comparison (e.g. *https://pubmed.ncbi.nlm.nih.gov/33824951/)1.

 These have been added and the citation above also included.
